# Roles of Dkk2 in the Linkage from Muscle to Bone during Mechanical Unloading in Mice

**DOI:** 10.3390/ijms21072547

**Published:** 2020-04-06

**Authors:** Naoyuki Kawao, Hironobu Morita, Shunki Iemura, Masayoshi Ishida, Hiroshi Kaji

**Affiliations:** 1 Department of Physiology and Regenerative Medicine, Kindai University Faculty of Medicine, Osakasayama 589-8511, Japan; kawao@med.kindai.ac.jp (N.K.); shunki0709@gmail.com (S.I.); masa-ishida@med.kindai.ac.jp (M.I.); 2 Department of Physiology, Gifu University Graduate School of Medicine, Gifu 501-1194, Japan; zunzunmorita@gmail.com

**Keywords:** Dkk2, muscle/bone interaction, mechanical unloading, osteopenia, muscle wasting

## Abstract

Mechanical unloading simultaneously induces muscle and bone loss, but its mechanisms are not fully understood. The interactions between skeletal muscle and bone have been recently noted. Although canonical wingless-related integration site (Wnt)/β-catenin signaling is crucial for bone metabolism, its roles in the muscle and bone interactions have remained unknown. Here, we performed comprehensive DNA microarray analyses to clarify humoral factors linking muscle to bone in response to mechanical unloading and hypergravity with 3 *g* in mice. We identified Dickkopf (Dkk) 2, a Wnt/β-catenin signaling inhibitor, as a gene whose expression was increased by hindlimb unloading (HU) and reduced by hypergravity in the soleus muscle of mice. HU significantly elevated serum Dkk2 levels and Dkk2 mRNA levels in the soleus muscle of mice whereas hypergravity significantly decreased those Dkk2 levels. In the simple regression analyses, serum Dkk2 levels were negatively and positively related to trabecular bone mineral density and mRNA levels of receptor activator of nuclear factor-kappa B ligand (RANKL) in the tibia of mice, respectively. Moreover, shear stress significantly suppressed Dkk2 mRNA levels in C2C12 cells, and cyclooxygenase inhibitors significantly antagonized the effects of shear stress on Dkk2 expression. On the other hand, Dkk2 suppressed the mRNA levels of osteogenic genes, alkaline phosphatase activity and mineralization, and it increased RANKL mRNA levels in mouse osteoblasts. In conclusion, we showed that muscle and serum Dkk2 levels are positively and negatively regulated during mechanical unloading and hypergravity in mice, respectively. An increase in Dkk2 expression in the skeletal muscle might contribute to disuse- and microgravity-induced bone and muscle loss.

## 1. Introduction

Sarcopenia is a pivotal disease concept linked to physical frailty and highly comorbid with osteoporosis in the elderly people worldwide [1]. Osteoporosis is associated with physical frailty due to decreased motility by osteoporotic vertebral and hip fractures. The clarification of the pathogenesis and mechanisms of sarcopenia and osteoporosis are therefore necessary to meet the clinical demand to prolong healthy aging.

Mechanical stress and unloading simultaneously affect skeletal muscle and bone. Mechanical unloading, such as long-term space flight and bed rest, rapidly reduces muscle and bone mass [1], indicating that mechanical stress is important for maintaining muscle and bone homeostasis. It has been noted that the interactions between muscle and bone might be involved in the mechanisms of sarcopenia and osteoporosis [1,2]. Muscle influences bone metabolism through the release of various humoral factors, myokines, such as myostatin, insulin-like growth factor-1, fibroblast growth factor 2, interleukin-6, osteoglycin, family with sequence similarity 5, member C (FAM5C), follistatin and irisin [1,2]. Previous studies indicated that exercise, mechanical stress and unloading regulate the secretion of myokines affecting bone metabolism [1,2]. Exercise reduces the expression of myostatin, a negative regulator of muscle mass and bone metabolism, in the skeletal muscle in humans and mice [2]. We previously reported that follistatin, an inhibitor of myostatin, contributes to bone mass increased by hypergravity in mice [3]. Moreover, irisin is involved in mechanical unloading-induced trabecular bone loss of mice in our study [4]. These findings suggest that mechanical stress and gravity change influence muscle and bone metabolism through the humoral factors linking muscle to bone. However, what kinds of humoral factors linking muscle to bone regulated by mechanical stress are important have not been fully understood.

Canonical wingless-related integration site (Wnt)/β-catenin signaling, a crucial regulator of bone metabolism, accelerates and suppresses osteoblastic bone formation and bone resorption, respectively [5]. The binding of the Wnt ligands to the dual receptor complex composed of Wnt co-receptors low-density lipoprotein receptor-related protein (LPR) 5/6 and frizzled proteins activates canonical Wnt/β-catenin signaling by inhibiting the phosphorylation of β-catenin and its proteasomal degradation [5]. Canonical Wnt/β-catenin signaling inhibitors, such as sclerostin, Dickkopfs (Dkks) and secreted frizzled related proteins (Sfrps), play key roles in maintaining musculoskeletal homeostasis. Sclerostin, mostly released from osteocytes, suppresses bone formation through competitive binding to LPR5/6, resulting in increased phosphorylation and degradation of β-catenin. Anti-sclerostin monoclonal antibody, such as romosozumab, exerts the potent therapeutic effects through an enhancement of bone formation and a suppression of bone resorption in patients with osteoporosis [6]. Dkk1 is expressed in several types of cells, including osteoblasts, osteocytes and osteoprogenitors, in bone tissues, and it antagonizes canonical Wnt/β-catenin signaling by binding to LRP5/6 [7,8]. Mukhopadhyay et al. revealed that Dkk1 is essential for embryonic head induction and limb morphogenesis in mice [9]. Heterozygous mutations in Dkk1 showed increased bone formation and bone mass without affecting bone resorption in mice [10]. Previous studies indicate that Dkk1 may play a crucial role in bone loss in pathophysiological conditions such as multiple myeloma, glucocorticoid excess and postmenopausal osteoporosis [11,12,13,14]. Moreover, mechanical loading suppresses Dkk1 expression in mice [15,16]. These findings suggest that Dkk1 is involved in the physiological regulation of bone metabolism, the pathogenesis of metabolic bone diseases and cancer bone metastasis. On the other hand, Chan et al. reported that the knockdown of Dkk2 restored abnormalities of alkaline phosphatase (ALP) activity and mineralization in osteoblasts from patients with osteoarthritis [17]. Li et al. revealed that Dkk2 plays roles in the terminal osteoblasts differentiation and mineralization in mice [18]. Moreover, an increase in Dkk1 and Dkk2 expression is required for osteoblast differentiation at late differentiation stage in osteoblastic KS483 cells [19]. These findings suggest that Dkk2 influences osteoblast phenotypes in mice, although the potent inhibitory effects of Dkk2 on canonical Wnt/β-catenin signaling is well known [20,21].

As for skeletal muscle, canonical Wnt/β-catenin signaling is required for embryonic skeletal myogenesis by regulating myogenic differentiation 1 (MyoD) and Myf5, the myogenic differentiation genes [22]. Wnt1 and Wnt3a ligands stimulate skeletal myogenesis within somites [23,24]. In postnatal myogenesis, canonical Wnt/β-catenin signaling influences the function of muscle satellite cells and skeletal muscle regeneration. Otto et al. revealed that canonical Wnt/β-catenin signaling induces the proliferation of satellite cells during adult skeletal muscle regeneration [25]. In contrast, Murphy et al. reported that transiently-activated Wnt/β-catenin signaling is not required but must be silenced for satellite cell function during muscle regeneration [26]. Taken together, these findings suggest that canonical Wnt/β-catenin signaling is important for muscle physiology as well as bone metabolism. However, the roles of canonical Wnt/β-catenin signaling-related factors in the linkage of muscle to bone and the effects of mechanical stress and gravity change on the interactions between muscle and bone remain unknown.

We previously showed that hindlimb unloading (HU) reduced muscle mass and tibial trabecular bone mineral density (BMD) in mice [4]. Moreover, hypergravity increased muscle mass and trabecular bone mineral content in the tibia adjusting for body weight in mice, as previously reported [27]. These data suggest the possibility that the interactions between skeletal muscle and bone metabolism may be involved in the effects of HU and hypergravity on muscle and bone in mice. In the present study, we therefore performed comprehensive DNA microarray analyses to clarify the mechanisms by which mechanical stress and gravity change affect muscle and bone metabolism through the humoral factors linking from muscle to bone.

## 2. Results

### 2.1. Microarray Analyses

Mechanical stress and gravity mainly affect postural muscles such as the mouse soleus muscles, which were chosen for the experiments presented here. 1 *g* or 3 *g* mice were exposed to 1 *g* or 3 *g* environments by centrifugation in a gondola-type rotating box for 4 weeks, respectively [3,27]. We performed comprehensive DNA microarray analyses in the soleus muscle between HU and control mice as well as 1 *g* and 3 *g* mice to explore the genes of humoral factors that are responsible for the interaction from muscle to bone affected by mechanical unloading or gravity change. The gene transcripts whose expression were upregulated (≥2.0) in the soleus muscle of HU mice, compared to control mice, were 815 genes. The gene transcripts whose expression was downregulated (≤0.5) in the soleus muscle of 3 *g* mice, compared to 1 *g* mice, were 517 genes. Among them, 34 genes were overlapped in the gene transcripts whose expression were upregulated by HU and downregulated by 3 *g* in the soleus muscle of mice, compared to those in control and 1 *g* mice (Table 1). Dkk2 was selected as the gene whose expression was the highest ratio of HU to control mice (13.3-fold) among humoral factors. In these comparative DNA microarray analyses, other Wnt signaling-related genes including Dkk1, Sfrp and Wnt ligands were not overlapped in the analyses of gene transcripts whose expression was upregulated (≥2.0) by HU and downregulated (≤0.5) by 3 *g* in the soleus muscle, compared to control and 1 *g* mice (Table 2).

### 2.2. Effects of HU and Hypergravity on Dkk2 in the Skeletal Muscle of Mice

**Figure 1:**
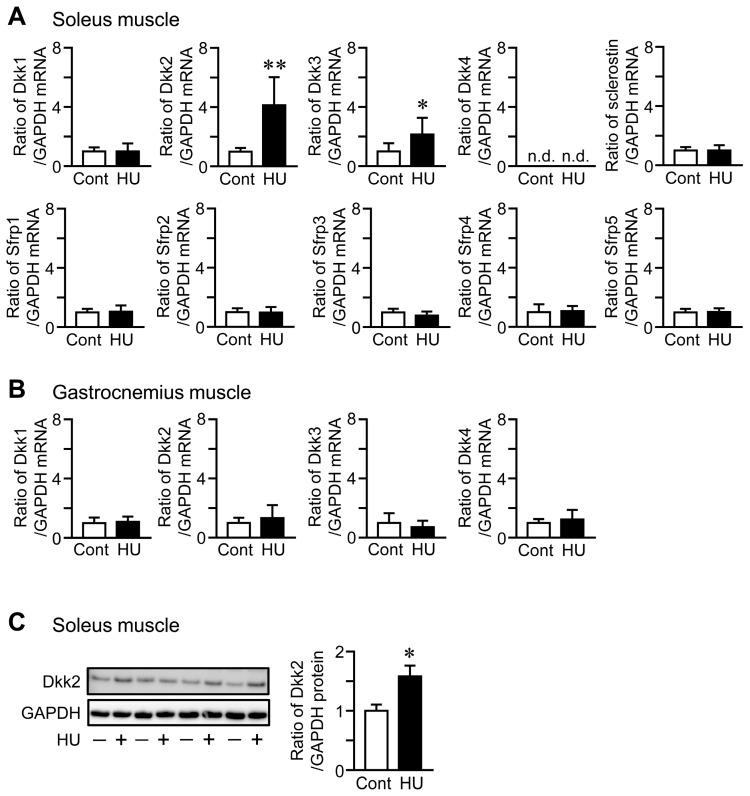
Effects of hindlimb unloading (HU) on the levels of canonical wingless-related integration site (Wnt)/β-catenin signaling-related factors in the skeletal muscle of mice. (**A**,**B**) Total RNA was extracted from the soleus (**A**) and gastrocnemius (**B**) muscles of control (Cont) mice and mice after HU for 3 weeks (*n* = 8 mice in each group). Then, real-time polymerase chain reaction (PCR) analysis of Dkk1, Dkk2, Dkk3, Dkk4, sclerostin, Sfrp1, Sfrp2, Sfrp3, Sfrp4, Sfrp5 or glyceraldehyde-3-phosphate dehydrogenase (GAPDH) was performed. Data represent the mean ± standard deviation (SD) of eight mice in each group. (**C**) Total protein was extracted from the soleus muscle of control mice (−) or mice after HU (+) for 3 weeks (*n* = 4 mice in each group). Then, Western blot analysis for Dkk2 or GAPDH was performed. The images represent experiments performed independently three times. Protein signals were quantified by densitometry and adjusted by the density of GAPDH. Data represent the mean ± SD of four mice in each group. ** *p* < 0.01, * *p* < 0.05 vs. control group (Mann-Whitney U test).

**Figure 2:**
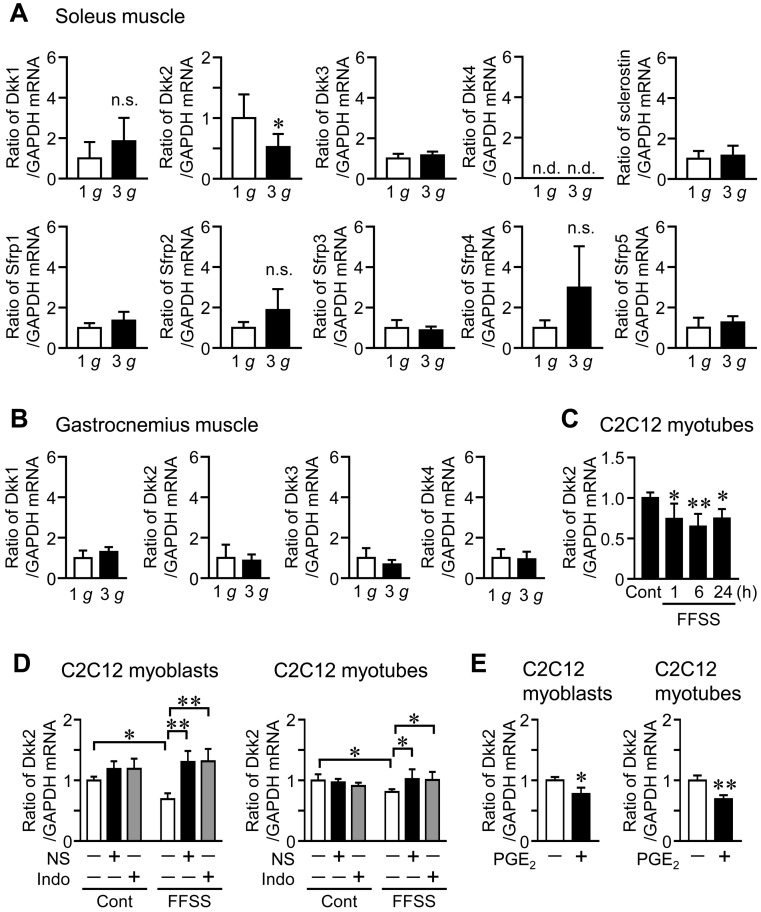
Effects of hypergravity on the levels of canonical Wnt/β-catenin signaling-related factors in the skeletal muscle of mice. (**A**,**B**) Total RNA was extracted from the soleus (**A**) and gastrocnemius (**B**) muscles of mice with exposure to 1 *g* or 3 *g* for 4 weeks (*n* = 8 mice in each group). Then, real-time PCR analysis of Dkk1, Dkk2, Dkk3, Dkk4, sclerostin, Sfrp1, Sfrp2, Sfrp3, Sfrp4, Sfrp5 or GAPDH was performed. Data represent the mean ± SD of eight mice in each group. * *p* < 0.05 vs. 1 *g* mice (Mann-Whitney U test). n.s., no significant difference. (**C**) Myoblastic C2C12 cells were cultured with Dulbecco’s Modified Eagle’s Medium (DMEM) and 2% horse serum for 5 days for differentiation into myotubes. Total RNA was extracted from C2C12 myotubes after exposure to fluid flow shear stress (FFSS) for 1, 6 or 24 h and static conditions (Cont) (*n* = 5 in each group). Then, real-time PCR analysis of Dkk2 or GAPDH was performed. Data represent the mean ± SD of five experiments in each group. ** *p* < 0.01 and * *p* < 0.05 vs. control group (Dunnett test). (**D**) Total RNA was extracted from C2C12 myoblasts or myotubes 6 h after exposure to FFSS in the presence (+) or absence (−) of 10 µM NS-398 (NS) or 1 µM indomethacin (Indo), and real-time PCR analysis of Dkk2 or GAPDH was performed (*n* = 6 in each group). Data represent the mean ± SD of six experiments in each group. ** *p* < 0.01 and * *p* < 0.05 (Tukey-Kramer test). (**E**) Total RNA was extracted from C2C12 myoblasts or myotubes cultured with (+) or without (−) 1 µM PGE_2_ for 24 h (*n* = 6 in each group). Real-time PCR analysis of Dkk2 or GAPDH was performed. Data represent the mean ± SD of six experiments in each group. ** *p* < 0.01 and ** *p* < 0.05 (Mann-Whitney U test).

HU significantly elevated Dkk2 mRNA and protein levels in the soleus muscle of mice, compared to control mice (Figure 1A,C). Although Dkk3 mRNA levels were increased in the soleus muscle of mice with HU compared to those of control mice, HU did not affect the mRNA levels of Dkk1, sclerostin, Sfrp1, Sfrp2, Sfrp3, Sfrp4 and Sfrp5 in the soleus muscle of mice (Figure 1A). No significant differences were observed in the mRNA levels of Dkk1, Dkk2, Dkk3 and Dkk4 in the gastrocnemius muscle between control mice and mice with HU (Figure 1B). Hypergravity significantly declined Dkk2 mRNA levels in the soleus muscle of mice, compared to those of mice with 1 *g*, although there are no significant differences in the mRNA levels of Dkk1, Dkk3, sclerostin, Sfrp1, Sfrp2, Sfrp3, Sfrp4 and Sfrp5 between 1 *g* and 3 *g* mice (Figure 2A). Hypergravity did not affect the mRNA levels of Dkk1, Dkk2, Dkk3 and Dkk4 in the gastrocnemius muscle of mice (Figure 2B).

### 2.3. Effects of Shear Stress on Dkk2 Expression in Muscle Cells in Vitro

Since mechanical unloading and hypergravity affected Dkk2 levels in muscle tissue of mice, we next examined the effects of fluid flow shear stress (FFSS), a biologically important mechanical stress, on Dkk2 mRNA levels in mouse C2C12 myotubes. Dkk2 mRNA levels were significantly reduced by FFSS for 1, 6 and 24 h in C2C12 myotubes (Figure 2C). Previous studies indicated that FFSS stimulates intracellular signaling such as cyclooxygenase (COX)-2 and prostaglandin E_2_ (PGE_2_) production [28,29]. We therefore examined the effects of COX inhibitors on FFSS-induced reduced Dkk2 expression in C2C12 cells. As shown in Figure 2D, a COX-2 selective inhibitor NS-398 and nonselective COX inhibitor indomethacin blunted reductions of Dkk2 mRNA levels during FFSS in C2C12 cells (Figure 2D). Moreover, PGE_2_ suppressed Dkk2 mRNA levels in C2C12 cells (Figure 2E).

### 2.4. Effects of HU and Hypergravity on Serum Dkk2 Levels

**Figure 3:**
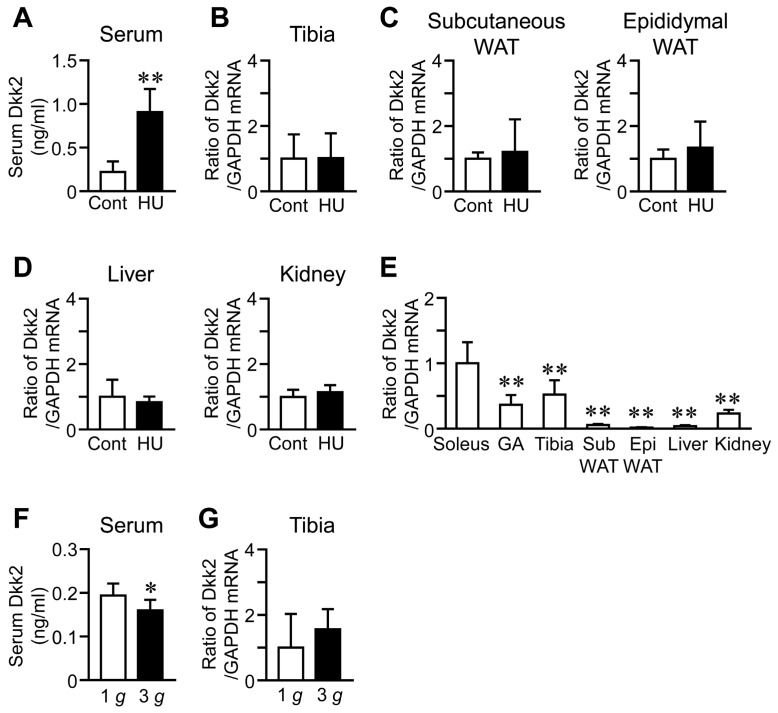
Effects of HU and hypergravity on serum as well as Dkk2 mRNA levels in the tibia, subcutaneous and epididymal white adipose tissues (WAT), liver and kidney. (**A**) Serum samples were collected from control (Cont) mice or mice with HU for 3 weeks (*n* = 8 mice in each group). Then, quantification of serum Dkk2 levels was performed. The data represent the mean ± SD of eight mice in each group. ** *p* < 0.01 vs. control group (Mann-Whitney U test). (**B**–**D**) Total RNA was extracted from the tibia (**B**), subcutaneous and epididymal WAT (**C**), liver and kidney (**D**) of control mice and mice with HU for 3 weeks (*n* = 8 mice in each group). Then, real-time PCR analysis of Dkk2 or GAPDH was performed. The data represent the mean ± SD of eight mice in each group. (**E**) Total RNA was extracted from the soleus and gastrocnemius (GA) muscles, tibia, subcutaneous (Sub) WAT, epididymal (Epi) WAT, liver and kidney of control mice (*n* = 8 mice in each group). Then, real-time PCR analysis of Dkk2 or GAPDH was performed. The data represent the mean ± SD of eight mice in each group. ** *p* < 0.01 vs. soleus muscle (Dunnett test). (**F**) Serum samples were collected from mice with exposure to 1 *g* or 3 *g* for 4 weeks (*n* = 8 mice in each group). Then, quantification of serum Dkk2 levels was performed. The data represent the mean ± SD of eight mice in each group. * *p* < 0.05 vs. 1 *g* group (Mann-Whitney U test). (**G**) Total RNA was extracted from the tibia of mice with exposure to 1 *g* or 3 *g* for 4 weeks (*n* = 8 mice in each group). Then, real-time PCR analysis of Dkk2 or GAPDH was performed. The data represent the mean ± SD of eight mice in each group.

We examined the effects of HU on serum Dkk2 levels to clarify whether Dkk2 is a humoral factor in response to mechanical unloading. Serum Dkk2 levels were significantly increased in mice with HU, compared to those of control mice (Figure 3A). In contrast, HU did not affect Dkk2 mRNA levels in the tibia, subcutaneous and epididymal white adipose tissues, liver and kidney (Figure 3B–D). The baseline Dkk2 mRNA levels were significantly higher in the soleus muscle than in the gastrocnemius muscle, tibia, white adipose tissues, liver and kidney of control mice (Figure 3E). Hypergravity significantly reduced serum Dkk2 levels, compared to 1 *g* mice, although it did not affect Dkk2 mRNA levels in the tibia of mice (Figure 3F,G).

### 2.5. Correlations between Dkk2 Levels and Bone Parameters during Mechanical Unloading

**Figure 4:**
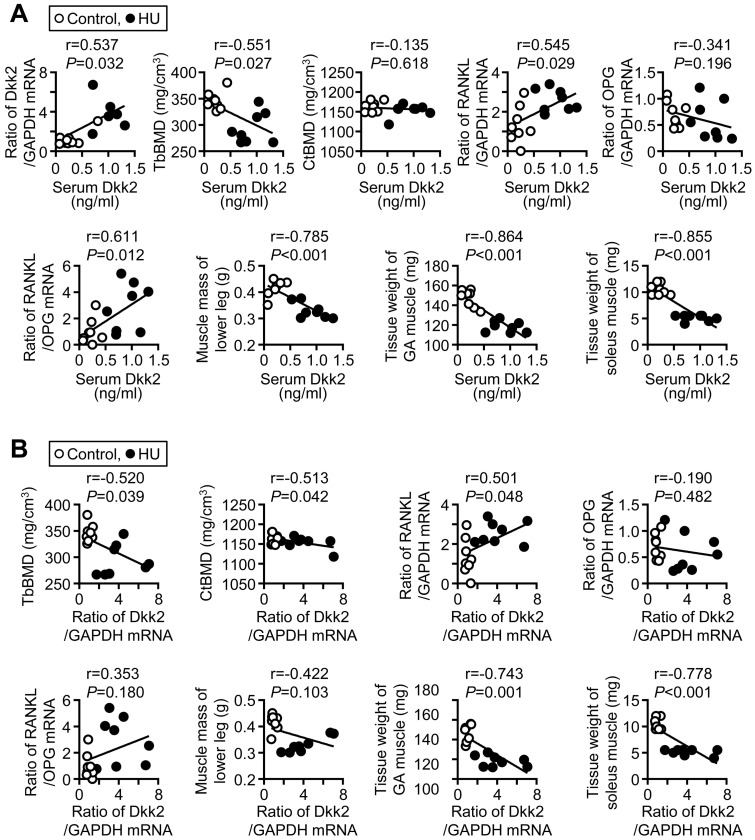
Relationship between Dkk2 and muscle/bone parameters in mice. (**A**) A simple regression analysis was performed between serum Dkk2 levels and Dkk2 mRNA levels in the soleus muscle, trabecular bone mineral density (TbBMD), cortical BMD (CtBMD), tibial nuclear factor-kappa B ligand (RANKL) mRNA levels, tibial osteoprotegerin (OPG) mRNA levels, ratio of RANKL/OPG mRNA in the tibia, muscle mass of lower legs or tissue weights of the gastrocnemius (GA) and soleus muscles in control mice and mice with HU for 3 weeks (*n* = 8 mice in each group). (**B**) Simple regression analysis was performed between Dkk2 mRNA levels in the soleus muscle and trabecular BMD, cortical BMD, tibial RANKL mRNA levels, tibial OPG mRNA levels, ratio of RANKL/OPG mRNA in the tibia, muscle mass of lower legs or tissue weights of the gastrocnemius (GA) and soleus muscles in control mice and mice with HU for 3 weeks (*n* = 8 mice in each group).

We next examined the relationships between bone parameters and serum Dkk2 levels as well as soleus Dkk2 mRNA levels in mice with HU and control mice by simple regression analysis. Serum Dkk2 levels were significantly and positively related to Dkk2 mRNA levels in the soleus muscle (Figure 4A). Serum Dkk2 levels were negatively related to trabecular, but not cortical, BMD, in the tibia of mice (Figure 4A). Serum Dkk2 levels were positively related to receptor activator of nuclear factor-kappa B ligand (RANKL) mRNA levels and the ratio of RANKL/osteoprotegerin (OPG) mRNA in the tibia of mice (Figure 4A). Dkk2 mRNA levels in the soleus muscle were significantly and negatively related to trabecular and cortical BMD in the control mice and mice with HU (Figure 4B). Moreover, Dkk2 mRNA levels in the soleus muscle were significantly and positively related to RANKL mRNA levels in the tibia (Figure 4B). Serum Dkk2 and soleus muscle Dkk2 mRNA levels were not related to the mRNA levels of osteogenic genes, including Runx2, Osterix, ALP and osteocalcin, in the tibia of control mice and mice with HU (Table S1). Serum Dkk2 and Dkk2 mRNA levels in the soleus muscle were significantly and negatively related to muscle mass of lower legs as well as tissue weight of the soleus and gastrocnemius muscles in control mice and mice with HU (Figure 4A,B).

### 2.6. Effects of Dkk2 on Muscle Cells

**Figure 5:**
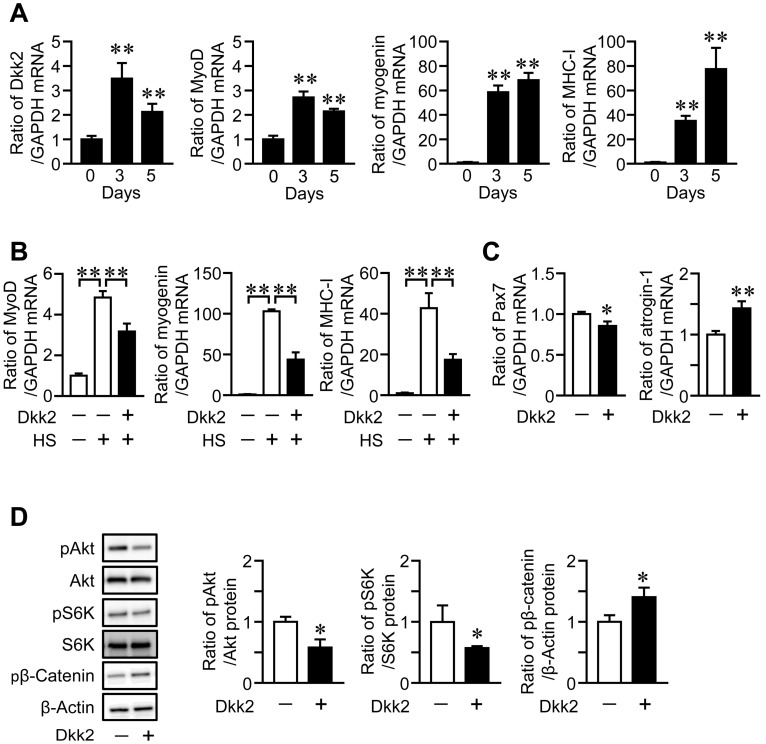
Effects of Dkk2 in muscle cells. (**A**) C2C12 cells were grown until confluent (day 0), and then cells were cultured with 2% horse serum for further 3 or 5 days. Total RNA was extracted from the cells, and real-time PCR analysis of myogenic differentiation 1 (MyoD), myogenin, myosin heavy chain (MHC)-I, Dkk2 or GAPDH was performed (*n* = 6 in each group). Data represent the mean ± SD of six experiments in each group. ** *p* < 0.01 vs. day 0 (Dunnett test). (**B**) Total RNA was extracted from confluent C2C12 cells (−) or C2C12 cells cultured in 2% horse serum (HS, +) with (+) or without (−) 500 ng/mL Dkk2 for 5 days (*n* = 6 in each group). Then, real-time PCR analysis of MyoD, myogenin, MHC-I or GAPDH was performed. Data represent the mean ± SD of six experiments in each group. ** *p* < 0.01 (Tukey-Kramer test). (**C**) Total RNA was extracted from C2C12 cells cultured with (+) or without (−) 500 ng/mL Dkk2 for 24 h (*n* = 5 in each group). Then, real-time PCR analysis of Pax7, atrogin-1 and GAPDH was performed. Data represent the mean ± SD of five experiments in each group. ** *p* < 0.01 and * *p* < 0.05 (Mann-Whitney U test). (**D**) Total protein was extracted from C2C12 cells cultured with (+) or without (−) 500 ng/mL Dkk2 for 24 h (*n* = 4 in each group). Then, Western blot analysis for phosphorylated Akt (pAkt), Akt, phosphorylated p70S6 kinase (pS6K), S6K, phosphorylated β-catenin (pβ-Catenin) and β-actin was performed. The images represent experiments performed independently three times. Protein signals of pAkt, pS6K and pβ-catenin were quantified by densitometry and adjusted by the density of Akt, S6K and GAPDH, respectively. Data represent the mean ± SD. * *p* < 0.05 vs. control group (Mann-Whitney U test).

We examined the expression of Dkk2 in mouse muscle cells during myogenic differentiation. The mRNA levels of Dkk2 and MyoD were increased, until the three-day culture of myoblastic C2C12 cells was completed with 2% horse serum, then, it declined after the five-day culture, although myogenin and myosin heavy chain (MHC)-I mRNA levels were elevated during the five-day cultures (Figure 5A). We next examined the effects of Dkk2 on muscle cells to clarify whether Dkk2 is involved in the mechanical unloading-induced muscle wasting in mice. Dkk2 significantly reduced the mRNA levels of MyoD, myogenin and MHC-I in C2C12 cells during myogenic differentiation (Figure 5B). Moreover, Dkk2 significantly suppressed the mRNA levels of Pax7, a myogenic differentiation-related factor, in C2C12 cells (Figure 5C). Dkk2 elevated atrogin-1, a muscle protein degradation-related factor, in C2C12 cells (Figure 5C). Since Akt/p70S6 kinase pathway plays key roles in protein synthesis in the skeletal muscle [3], we next examined the effects of Dkk2 on phosphorylation of Akt and p70S6 kinase in C2C12 cells. Dkk2 reduced phosphorylation of Akt and p70S6 kinase in C2C12 cells (Figure 5D). Dkk2 facilitated the phosphorylation of β-catenin in C2C12 cells (Figure 5D).

### 2.7. Effects of Dkk2 on Bone Cells

**Figure 6:**
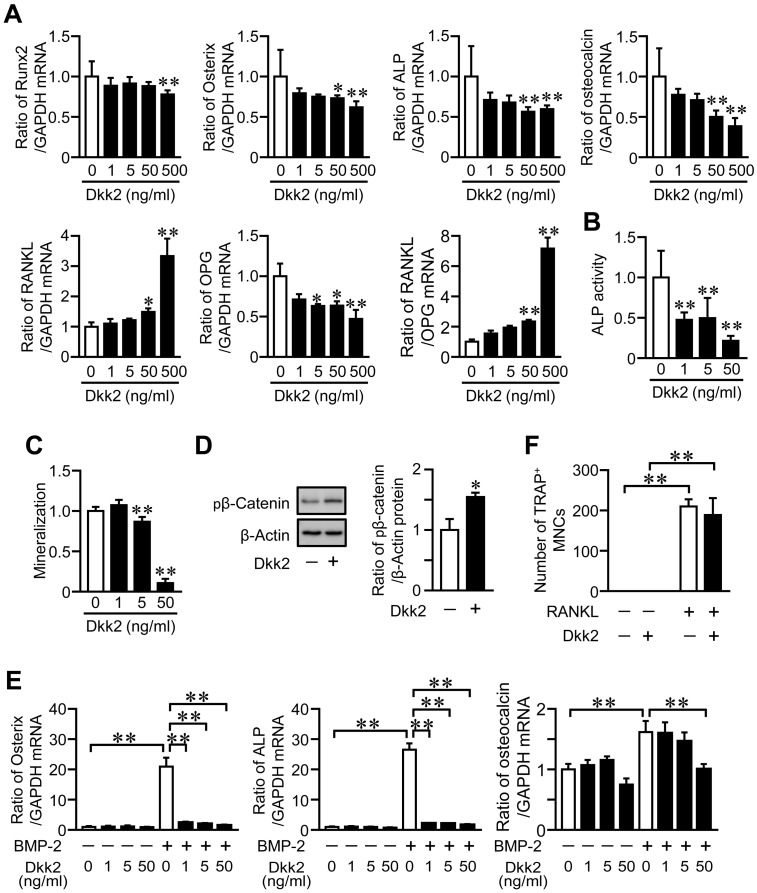
Effects of Dkk2 on the osteoblast phenotypes and osteoclast formation. (**A**) Total RNA was extracted from mouse osteoblasts cultured with the indicated concentrations of Dkk2 for 24 h, and real-time PCR analysis of Runx2, Osterix, ALP, osteocalcin, RANKL, OPG or GAPDH was performed (*n* = 6 in each group). Data represent the mean ± SD of six experiments in each group. ** *p* < 0.01 and * *p* < 0.05 vs. control group (Dunn test). (**B**) ALP activity was measured in confluent mouse osteoblasts cultured with the indicated concentrations of Dkk2 for 24 h as described in Materials and Methods (*n* = 6 in each group). Data represent mean ± SD of six experiments in each group. ** *p* < 0.01 vs. control group (Dunnett test). (**C**) Mouse osteoblasts were cultured with 10 mM β-glycerophosphate in the presence of the indicated concentrations of Dkk2 for 3 weeks (*n* = 5 in each group). Mineralization was determined by Alizarin red staining as described in Materials and Methods. Data represents mean ± SD of five experiments in each group. ** *p* < 0.01 vs. control group (Dunnett test). (**D**) Total protein was extracted from mouse osteoblasts cultured with (+) or without (−) 500 ng/mL Dkk2 for 24 h (*n* = 4 in each group). Then, Western blot analysis for phosphorylated β-catenin (pβ-Catenin) and β-actin was performed. The images represent experiments performed independently three times. Protein signals were quantified by densitometry and adjusted by the density of GAPDH. Data represent the mean ± SD. * *p* < 0.05 vs. control group (Mann-Whitney U test). (**E**) Total RNA was extracted from ST2 cells cultured with (+) or without (−) 200 ng/mL BMP-2 in the presence of the indicated concentrations of Dkk2 for 72 h, and real-time PCR analysis of Osterix, ALP, osteocalcin or GAPDH was performed (*n* = 5 in each group). Data represent the mean ± SD of five experiments in each group. ** *p* < 0.01 (Tukey-Kramer test). (**F**) RAW264.7 cells were cultured with (+) or without (−) 75 ng/mL RANKL in the presence (+) or absence (−) of 500 ng/mL Dkk2 for 4 days (*n* = 6 in each group). The cells were stained with tartrate-resistant acid phosphatase (TRAP) staining, and the number of TRAP-positive multinucleated cells (TRAP^+^ MNCs) was counted in each well. The data represent the mean ± SD of six experiments. ** *p* < 0.01 (Tukey-Kramer test).

We examined the effects of Dkk2 on the osteoblast phenotypes. Dkk2 significantly and dose-dependently reduced the mRNA levels of Runx2, Osterix, ALP and osteocalcin in mouse primary osteoblasts (Figure 6A). Dkk2 significantly elevated RANKL mRNA levels as well as the ratio of RANKL/OPG mRNA in these cells, although it decreased OPG mRNA levels (Figure 6A). Moreover, Dkk2 significantly suppressed ALP activity and mineralization in mouse osteoblasts (Figure 6B,C), and these effects were dose-dependent. Dkk2 enhanced the phosphorylation of β-catenin in osteoblasts (Figure 6D). We examined the effects of Dkk2 on differentiation of mesenchymal cells into osteoblasts. Dkk2 significantly suppressed the levels of Osterix, ALP and osteocalcin enhanced by bone morphogenetic protein-2 (BMP-2) treatment in mouse mesenchymal ST2 cells (Figure 6E). We finally investigated the effects of Dkk2 on osteoclast formation from mouse monocytic RAW264.7 cells. Dkk2 did not affect the osteoclast formation from RAW264.7 cells in the presence of RANKL (Figure 6F).

## 3. Discussion

In the present study, Dkk2 was extracted as the gene whose expression was increased and decreased in the soleus muscle of mice with mechanical unloading and hypergravity, respectively, using comparative DNA microarray analyses. In simple regression analyses, both serum and soleus muscle Dkk2 levels were negatively and positively related to tibial trabecular BMD and RANKL expression. Dkk2 decreased the differentiation, ALP activity and mineralization in mouse osteoblasts, although it enhanced RANKL expression.

The importance of Wnt/β-catenin signaling inhibitors, such as sclerostin, Dkks and Sfrps, in regulation of bone metabolism has been demonstrated by numerous studies in humans and genetically modified mice [5,7,8,9,10,11,12,13,14,18]. An activation of canonical Wnt/β-catenin signaling exerts positive effects on bone homeostasis through an enhancement of osteoblast differentiation and proliferation [5]. In the present study, we showed that trabecular BMD was negatively related to serum Dkk2 levels and muscle Dkk2 expression in mice with and without mechanical unloading. Moreover, serum Dkk2 levels were positively related to RANKL expression and the ratio of RANKL/OPG mRNA in the bone tissues. In in vitro experiments, Dkk2 suppressed the differentiation, ALP activity and mineralization as well as an enhancement of β-catenin phosphorylation, a key step of canonical Wnt/β-catenin signaling inhibition, in mouse osteoblasts. Moreover, Dkk2 enhanced and reduced the expressions of RANKL and OPG in these cells, respectively, although it did not affect osteoclast formation from mouse monocytic cells in the presence of RANKL. These results suggest that increased levels of circulating Dkk2 contributes to bone loss during mechanical unloading as a humoral factor linking muscle to bone, although the lack of effect of hindlimb unloading to Dkk2 in the tibia and the impact of Dkk2 on bone cells might be related to the high homology of Dkk2 to Dkk1.

Li et al. reported that Dkk2 deficiency exhibits decreased bone mass in mice [18]. In that study, Dkk2 deficiency suppressed the expression of late stage osteoblast differentiation genes, such as osteocalcin in the osteoblasts of mice, and Dkk2 overexpression in osteoblasts enhanced mineralization [18]. Moreover, van der Horst et al. showed that a reduction in Dkk2 by small interfering RNA significantly reduced ALP activity and formation of mineralized bone nodules in osteoblastic KS483 cells [19]. These findings seemed to be contradictory to the evidence that Dkk2 is a potent antagonist for canonical Wnt/β-catenin signaling in cells [5,21]. However, Li et al. revealed that the effects of Dkk2 on osteoblastic differentiation and mineralization at late stages seem to be mediated by the mechanisms dependently and independently of its canonical Wnt/β-catenin signaling antagonistic effects [18]. Previous studies showed that Dkk2 activates canonical Wnt/β-catenin signaling partly mediated by the expression of kremen2 [20,30]. Our present study showed that Dkk2 treatment suppresses the osteoblast phenotypes followed with enhanced phosphorylation of β-catenin in mouse osteoblasts. Moreover, it enhanced and reduced the expressions of RANKL and OPG in these cells, respectively. These findings suggest that the inhibitory effects of exogenous Dkk2, as a humoral factor linking muscle to bone, on the osteoblast phenotypes are mediated by an inhibition of canonical Wnt/β-catenin signaling in mouse osteoblasts. Taken together, endogenous and exogenous Dkk2 might differently affect osteoblasts in mice.

Bone is a mechanical stress-sensing organ. Although the mechanisms of mechanical stress-sensing system have not been fully determined, a Wnt/β-catenin signaling inhibitor, sclerostin, is involved in the osteocyte-mediated regulation of bone metabolism in response to mechanical stress [5]. In the present study, mechanical unloading increased serum and muscle Dkk2 levels in mice, and hypergravity decreased them. Moreover, shear stress reduced Dkk2 expression in C2C12 cells. These findings suggest that canonical Wnt/β-catenin signaling is a crucial mediator of the mechanical stress-induced changes in muscle/bone relationships. Previous studies revealed that shear stress stimulates several intracellular signaling pathways, such as BMP/Smad signaling, phosphatidylinositol 3-kinase/Akt/p70S6 and arachidonate cascade [4,28,29]. In the present study, nonselective COX and selective COX-2 inhibitors blocked a reduction of Dkk2 expression during shear stress in C2C12 cells. Moreover, PGE_2_ decreased Dkk2 expression in C2C12 cells. These results indicate that PGE_2_ might be involved in a reduction of Dkk2 during mechanical stress in muscle cells. Further studies are necessary to clarify the precise mechanisms by which mechanical stress regulates Dkk2 expression in the skeletal muscle.

Although controversy exists regarding the roles of canonical Wnt/β-catenin signaling in muscle regeneration and muscle progenitor cell functions, canonical Wnt/β-catenin signaling is crucial for muscle development as well as embryonic and fetal limb myogenesis [22]. Kuroda et al. reported that muscle specific transgenic expression of β-catenin enhances slow-twitch muscle fibrogenesis in mice [31]. Hutcheson et al. revealed that overexpression of β-catenin in Pax7-positive lineage cells results in a reduced number of slow-twitch fibers during fetal myogenesis [32]. These findings suggest that canonical Wnt/β-catenin signaling positively regulates myogenesis and muscle mass. In the present study, Dkk2 suppressed myogenic differentiation as well as protein synthesis-related signaling, Akt and p70S6 kinase, and it increased the expression of protein degradation-related factor, atrogin-1, in C2C12 cells. Moreover, we showed that serum and muscle Dkk2 levels were negatively related to the muscle mass of the lower legs as well as tissue weights of the soleus and gastrocnemius muscles in mice. These results suggest that Dkk2 contributes to muscle wasting through inhibition of myogenesis, enhancement of muscle protein degradation and suppression of muscle protein synthesis during mechanical unloading. Yin et al. revealed that skeletal muscle Dkk3 is involved in age-related muscle atrophy through enhancement of E3 ubiquitin ligase protein degradation system in mice [33]. In the present study, mechanical unloading elevated Dkk3 expression in the soleus muscle of mice, although hypergravity did not affect them. We therefore could not exclude a possibility that Dkk3 might be involved in muscle atrophy induced by mechanical unloading in mice.

Dkk2 was identified as a humoral factor upregulated (13.3-fold) by mechanical unloading and downregulated (0.2-fold) by hypergravity in the soleus muscle of mice using the comparative DNA microarray analyses in this study. As for the other factors, arrestin domain-containing protein 2 (Arrdc2) contains PPXY motifs and binds to the WW domain-containing protein such as E3 ubiquitin ligases [34]. Although the functions of Arrdc2 have not yet been elucidated in detail, previous studies showed that Arrdc2 levels in the skeletal muscle are upregulated by catabolic stimuli such as fasting, androgen deficiency and glucocorticoid excess [35,36], which are consistent with our findings. Chac1, cation transport regulator 1 (Chac1) was identified as an ER stress inducible gene and degrades glutathione acting as γ-glutamyl cyclotransferase [37]. Nicotinamide riboside kinase 2 (Nmrk2) is involved in the synthesis of nicotinamide mononucleotide, which is an important intermediate in the biosynthesis of nicotinamide adenine dinucleotide [38]. Nmrk2 is abundantly expressed in the skeletal muscle and contributes to changes of myofiber type, fast fibers diameter and myokine levels after endurance exercise in aged mice [38]. Dkk2 contains two cysteine-rich domains, which were conserved regions in Dkk family, separated by a linker region [39]. Dkk2 expresses in many human tissues, including skeletal muscle, heart, lung and brain [39]. Although Dkk1, Dkk2 and Dkk4 can bind Lrp5/6 to suppress canonical Wnt/β-catenin signaling, Dkk3 is unable to bind Lrp5/6 [33]. Previous studies suggest that Dkks plays key roles in embryonic cell fate and bone metabolism [40]. In our data, mechanical unloading significantly enhanced the expression of Dkk2 in the soleus muscle and serum Dkk2 levels in mice, although it did not affect Dkk2 expression in the tibia, white adipose tissues, liver and kidney. We therefore speculated that an elevation in serum Dkk2 level induced by mechanical unloading might be mainly due to the enhanced secretion from skeletal muscle tissues in mice, although the further studies using the skeletal muscle-specific Dkk2-deleted mice is necessary to confirm our hypothesis.

Accumulating evidence suggest that canonical Wnt/β-catenin signaling is an important regulator of muscle and bone homeostasis [5,22]. Osteocytes play a sensor of mechanical stress in bone, and they secrete several humoral factors, such as sclerostin and Dkk1 [41]. In the present study, we revealed that Dkk2 contributes to bone loss during mechanical unloading and gravity change as a factor linking muscle to bone. These findings suggest that regulators of canonical Wnt/β-catenin signaling might be key factors to connect skeletal muscle and bone. Although anti-sclerostin monoclonal antibody is expected for a novel therapeutic drug for the treatment of osteoporosis [6], there are no effective drugs for the treatment of both sarcopenia and osteoporosis at the present time. Our data suggest that Dkk2 secreted from the skeletal muscle is involved in bone loss and muscle atrophy induced by mechanical unloading in mice. Taken together, Dkk2 might be a target for the development of a therapeutic drug for both sarcopenia and osteoporosis due to disuse.

There are several limitations in this study. First, we used 12- and 8-week-old mice for hindlimb unloading and hypergravity experiments, respectively, in the present study. We therefore cannot rule out the possibility that different age of the mice might influence the regulation of Dkk2 by mechanical unloading and hypergravity. Second, sex difference might exist for the roles of Dkk2 in the linkage from muscle to bone during mechanical unloading in mice, because only male mice were employed in the present study. Third, we suggested that PGE_2_ might be involved in a reduction of Dkk2 during mechanical stress in muscle cells using only the mRNA level data, but not protein level. Future experiments will be necessary to confirm our suggestion using Dkk2 specific antibody with higher quality for Western blot.

In conclusion, we provide novel evidence that Dkk2 expression is increased and decreased by mechanical unloading and hypergravity in the skeletal muscle of mice, respectively. The present study suggests that an increase in Dkk2 expression in the skeletal muscle might be involved in the mechanical unloading-induced bone loss, presumably through the inhibition of osteoblast differentiation and bone resorption via RANKL induction.

## 4. Materials and Methods

### 4.1. Materials

Anti-Dkk2 antibody was purchased from Proteintech (Cat. No. 21051-1-AP, Rosemont, IL, USA). Anti-phosphorylated Akt (Cat. No. 4060), anti-Akt (Cat. No. 4691), anti-phosphorylated p70S6 kinase (Cat. No. 9234), anti-p70S6 kinase (Cat. No. 2708), anti-glyceraldehyde-3-phosphate dehydrogenase (GAPDH, Cat. No. 5174) and anti-β-actin (Cat. No. 4970) antibodies were obtained from Cell Signaling Technology (Danvers, MA, USA). Anti-phosphorylated β-catenin (pSer^33^/pSer^37^) antibody was obtained from Sigma (Cat. No. C4231, Saint Louis, MO, USA). Recombinant Dkk2 was purchased from ReliaTech GmbH (Wolfenbuttel, Germany). NS-398 and RANKL were obtained from Wako Pure Chem (Osaka, Japan). Indomethacin and PGE_2_ were purchased from Sigma and Cayman Chemical (Ann Arbor, MI, USA), respectively. BMP-2 was provided by Pfizer Inc. (Groton, CT, USA).

### 4.2. Hindlimb Unloading Experiments

Male C57BL/6J mice were purchased from CLEA Japan (Tokyo, Japan). Mice, 12-week-old, were randomly divided into two groups: control (*n* = 8) and HU (*n* = 8). The mouse tail was suspended for 3 weeks using a tail clip (Yamashita Giken, Tokushima, Japan) for HU, as previously described [4,42]. During HU, the mice could move by own forelimbs. The mice were euthanized with excess isoflurane 3 weeks after tail suspension. All mice were fed *ad libitum* with food and water. The experiments were performed according to the guidelines of the National Institutes of Health and the institutional rules for the use and care of laboratory animals at Kindai University. The study protocols were approved by the Experimental Animal Welfare Committee of Kindai University (KAME-27-029, 27 July 2015).

### 4.3. Hypergravity Experiments

Male C57BL/6J mice were purchased from Chubu Kagaku Shizai (Nagoya, Japan). The mice were randomly divided into two groups: 1 *g* (*n* = 8) and 3 *g* (*n* = 8). The 8-week-old mice were exposed to 1 *g* or 3 *g* environments induced by centrifugation in a custom-made, gondola-type rotating box for 4 weeks, as described previously [3,27]. To obtain the 3 *g* environment, 41 rpm was applied. The centrifuge was stopped for 30 min to clean the cages and to supply food and water twice a week. Mice with exposure to 1 *g* and 3 *g* were kept in the same experimental room. All mice were fed *ad libitum* with food and water during the experiments. The experiments were performed according to the guidelines of the National Institutes of Health and the “Guiding Principles for Care and Use of Animals in the Field of Physiological Science” set by the Physiological Society of Japan. The experiments were approved by the Animal Research Committees of Gifu University (27-79, 31 March 2015).

### 4.4. Microarray Analyses

Microarray analyses were performed, as described previously [43,44]. Total RNA was extracted from the soleus muscle of mice with or without HU for 3 weeks as well as mice exposed to 1 *g* or 3 *g* for 4 weeks using RNeasy Mini Kit (Qiagen, Hilden, Germany). cDNA was synthesized using a High-Capacity cDNA Reverse Transcription Kit (Applied Biosystems, Foster, CA, USA). As for mice with or without HU, hybridization samples were prepared and processed using the GeneChip Hybridization, Wash and Stain kit (Affymetrix, Thermo Fishers Scientific) according to the manufacture’s protocol. Clariom S Array, Mouse (Affymetrix, Thermo Fishers Scientific) was used to compare gene expression. The scanned raw data were analyzed using Transcriptome Analysis Console (TAC) 4.0 software (Affymetrix, Thermo Fishers Scientific). As for mice exposed to 1 *g* or 3 *g*, hybridization samples were prepared and processed according to the GeneChip Expression Analysis Technical Manual. The Affymetrix Murine Genome 430 2.0 set was used to compare gene expression. Data were analyzed using the GeneChip Operating Software Version 1.4 (Affymetrix 690036) according to the GeneChip Expression Analysis Fundamentals, no.701190 Rev.4. Using DNA MicroArray Viewer (Kurabo, Osaka, Japan), the fold changes in expression between two groups were calculated.

### 4.5. Real-Time Polymerase Chain Reaction (PCR)

Total RNA was isolated from mouse tissues and cells using an RNeasy Mini Kit (Qiagen), as previously described [3]. The tibial bone tissues included bone marrow. A High-Capacity cDNA Reverse Transcription Kit (Applied Biosystems) was used for reverse transcription reaction. SYBR Green-based real-time PCR was performed using an ABI PRISM 7900HT (Applied Biosystems) with a Fast SYBR Green Master Mix (Applied Biosystems). Each PCR primer set is shown in Table S2. The specific mRNA amplification of the target was determined as the Ct value, which was followed by normalization with the GAPDH levels.

### 4.6. Western Blot Analysis

Mouse tissues and cells were lysed in a lysis buffer (Cell Signaling Technology) with protease inhibitors (Wako Pure Chem.). Western blot analysis was performed as described previously [3]. Briefly, after protein levels were quantified using a bicinchoninic acid assay reagent (Pierce, Rockford, IL, USA), equal amounts of protein aliquots were separated by electrophoresis on 4%–20% Mini-Protean Tris-Glycine extended precast gels (BioRad Laboratories, Hercules, CA, USA). The proteins were transferred to a polyvinyl difluoride membrane (Millipore, Bedford, MA, USA). Then, the membrane was blocked with 3% skim milk or bovine serum albumin, and subsequently incubated with each primary antibody. After the membrane was incubated with an appropriate secondary antibody, the immunolabelled proteins were visualized using the ECL select detection system (GE Healthcare, Tokyo, Japan).

### 4.7. Blood Chemistry

Serum Dkk2 levels were measured using an enzyme-linked immunosorbent assay kit for mouse Dkk2 (Cat. No. LS-F13151, LifeSpan BioSciences, Seattle, WA, USA).

### 4.8. Quantitative Computed Tomography (QCT) Analysis

QCT analysis was performed using an X-ray CT system in vivo (Latheta LCT-200; Hitachi Aloka Medical, Tokyo, Japan) in mice at 3 and 4 weeks after HU and 3 *g* condition, respectively, according to the guidelines of the American Society for Bone and Mineral Research [45], as described previously [46]. After mice were anesthetized with 2% isoflurane, CT images were acquired using the following parameters: 50 kVp tube voltage, 500 µA tube current, 3.6 ms integration time, 48 mm axial field of view and 24 µm isotropic voxel size for analysis of the tibia or 48 × 48 × 192 µm voxel size for analysis of lower legs. Regions of interest were defined as 1680 µm (70 slices) segments from 96 µm distal to the end of the proximal growth plate towards the diaphysis for assessment of trabecular BMD and as 2160 µm (90 slices) segments of the mid-diaphysis of the tibia for assessment of cortical BMD. The region of interest was defined as the segment from the proximal end to the distal end of the tibia for the assessment of muscle mass in the lower legs. BMDs and muscle mass were analyzed using LaTheta software (version 3.40, Hitachi Aloka Medical, Tokyo, Japan).

### 4.9. Cell Culture

Mouse myoblastic C2C12 cells and mouse monocytic RAW264.7 cells were obtained from ATCC (Manassas, VA, USA) and cultured in Dulbecco’s Modified Eagle’s Medium (DMEM; Wako Pure Chem.) with 10% fetal bovine serum (FBS) and 1% penicillin/streptomycin. Mouse mesenchymal ST2 cells (RIKEN Cell Bank, Tsukuba, Japan) were cultured in Minimum Essential Medium Alpha Modification (α-MEM; Wako Pure Chem.) with 10% FBS and 1% penicillin/streptomycin. The medium was changed twice a week.

### 4.10. Fluid Flow Shear Stress (FFSS)

After C2C12 cells were reaching confluence, the cells were cultured in DMEM with 2% horse serum and 1% penicillin/streptomycin for 5 days to facilitate myogenic differentiation. The cells were placed on an orbital shaker (KS 260 basic, IKA, Tokyo, Japan) and exposed to a nonpulsatile FFSS of approximately 7.6 dyn/cm^2^ in an incubator at 37 °C, as described previously [3,4].

### 4.11. Preparation of Primary Osteoblasts

Primary osteoblasts were obtained from the calvaria of three-day-old C57BL/6J mice, as described previously [47]. Briefly, after the mice were euthanized with excess isoflurane, the calvaria was removed and digested four times with 1 mg/mL collagenase and 0.25% trypsin for 20 min at 37 °C. Cells were collected from second, third and fourth digestions, and grown in α-MEM with 10% FBS and 1% penicillin/streptomycin.

### 4.12. ALP Activity

ALP activity in osteoblasts was analyzed, as described previously [48]. After reaching confluence, ALP activity was determined using a Lab assay ALP kit (Wako Pure Chem.), according to the manufacturer’s instructions. The absorbance was measured at 405 nm using a microplate reader and normalized to the total protein content. ALP activity was defined as [units/total protein (μg)].

### 4.13. Mineralization of Calvarial Osteoblasts

Mineralization of calvarial osteoblasts was determined by Alizarin Red staining, as described previously [47]. After reaching confluence, osteoblasts were cultured in α-MEM with 10% FBS, 10 mM β-glycerophosphate and 1% penicillin/streptomycin for 3 weeks. The cells were fixed with ice-cold 70% ethanol and stained with Alizarin Red S solution. For quantification, the stained cells were destained with 10% cetylpyridinium chloride, after which the extracted stain was transferred to a 96-well plate and the absorbance was measured at 570 nm.

### 4.14. Osteoclast Formation

Osteoclast formation was induced as described previously [3,43]. Briefly, RAW264.7 cells were seeded in a 96-well plate and cultured in α-MEM with 10% FBS, 1% penicillin/streptomycin and 75 ng/mL RANKL for 4 days. Detection of osteoclasts was performed using a tartrate-resistant acid phosphatase (TRAP) staining kit (Wako Pure Chem.), and number of TRAP-positive multinucleated cells (MNCs) was counted in each well.

### 4.15. Statistical Analysis

Data are expressed as the mean ± the standard deviation (SD). The results represent experiments performed independently three times. Statistical significance was evaluated using the non-parametric Mann-Whitney U test for comparisons of two groups. Normality of distribution was tested using Shapiro–Wilk test for multiple comparisons. Parametric one-way or two-way analysis of variance followed by the Dunnett test or Tukey-Kramer test were performed for multiple comparisons when data presented a normal distribution. Non-parametric Dunn test was performed when data did not present a normal distribution for multiple comparisons. Simple regression analysis was performed by the Pearson test. The significance level was set at *p* < 0.05. All statistical analyses were performed using GraphPad PRISM 7.00 software (GraphPad Software, Inc., La Jolla, CA, USA).

## Figures and Tables

**Table 1:** Gene transcripts in the soleus muscle of mice with HU versus control mice as well as 3 *g* mice versus 1 *g* mice.

Gene	Gene Accession Number	Fold Change	
		HU (≥2.0)	3 *g* (≤0.5)
Arrdc2	NM_027560	111.3	0.3
Chac1	NM_026929	18.7	0.4
Nmrk2	NM_027120	13.5	0.4
Dkk2	NM_020265	13.3	0.2
Cebpd	NM_007679	12.9	0.4
Mylk4	NM_001166030	11.5	0.3
Slc25a25	ENSMUST00000028160	8.1	0.3
Mafb	NM_010658	7.1	0.4
Gadd45g	NM_011817	6.6	0.4
Prkag3	NM_153744	6.4	0.4
Gm3893	NR_033506	6.4	−2.1
Ucp3	NM_009464	3.6	0.5
Nrip1	NM_173440	3.6	0.5
Neto2	NM_001081324	3.5	0.5
Tob2	ENSMUST00000050467	3.2	0.3
Fam78a	NM_175511	3.1	0.4
4933409K07Rik	NR_033123	2.9	0.4
Gm14347	NM_001085543	2.9	0.4
Igfbp5	NM_010518	2.5	0.5
Lrrc46	NM_027026	2.4	0.2
Dusp14	NM_019819	2.4	0.5
Trim7	NM_053166	2.3	0.5
Gm14625	XM_030251462	2.2	0.04
Eid3	NM_025499	2.2	0.14
Tmem225	NM_029379	2.1	0.2
Smox	NM_001177833	2.1	0.4
Gprc5c	NM_001110337	2.1	0.5
Hlf	NM_172563	2.1	0.4
Per2	NM_011066	2.1	0.5
Slc25a33	NM_027460	2.1	0.5
Fam107a	NM_183187	2.1	0.4
Nr4a1	NM_010444	2.1	0.3
Gm3417	NM_001123368	2.0	9 × 10^−3^
Dnah7c	ENSMUST00000178395	2.0	0.2

Data represent fold change (*n* = 1).

**Table 2:** Gene transcripts of Wnt signal-related factors in the soleus muscle of mice with HU versus control mice as well as 3 *g* mice versus 1 *g* mice.

Gene	Gene Accession Number	Fold Change	
		HU	3 *g*
Dkk1	NM_010051	−1.0	0.9
Dkk2	NM_020265	13.3	0.2
Dkk3	NM_015814	−1.2	0.9
Dkk4	NM_145592	−1.1	2.5
Sfrp1	NM_013834	−2.0	1.1
Sfrp2	NM_009144	−1.7	0.8
Frzb (Sfrp3)	NM_011356	1.3	0.3
Sfrp4	NM_016687	−21.4	0.9
Sfrp5	NM_018780	2.5	1.9
Wnt1	NM_021279	−1.2	0.7
Wnt2	NM_023653	−1.3	0.7
Wnt2b	NM_009520	1.8	6.1
Wnt3	NM_009521	1.1	0.3
Wnt3a	NM_009522	−1.5	0.3
Wnt4	NM_009523	3.0	0.9
Wnt5a	NM_009524	−1.2	1.1
Wnt5b	NM_009525	1.2	1.5
Wnt6	NM_009526	1.1	0.9
Wnt7a	NM_009527	1.1	1.1
Wnt7b	NM_009528	−1.0	0.7
Wnt8a	NM_009290	−1.1	8.6
Wnt8b	NM_011720	−1.0	2.3
Wnt9a	NM_139298	−1.4	1.1
Wnt9b	NM_011719	1.1	2.6
Wnt10a	NM_009518	−1.1	1.1
Wnt10b	NM_011718	−1.2	2.3
Wnt11	NM_009519	1.0	0.9
Wnt16	NM_053116	1.0	1.1

Dkk, Dickkopf; Sfrp, secreted frizzled related protein. Data represent fold change (*n* = 1).

## Supplementary Materials

Supplementary materials can be found at https://www.mdpi.com/1422-0067/21/7/2547/s1.

## References

[1] Kawao N., Kaji H. (2015). Interactions between muscle tissues and bone metabolism. J. Cell Biochem..

[2] Kaji H. (2016). Effects of myokines on bone. Bonekey Rep..

[3] Kawao N., Morita H., Obata K., Tatsumi K., Kaji H. (2018). Role of follistatin in muscle and bone alterations induced by gravity change in mice. J. Cell Physiol..

[4] Kawao N., Moritake A., Tatsumi K., Kaji H. (2018). Roles of irisin in the linkage from muscle to bone during mechanical unloading in mice. Calcif. Tissue Int..

[5] Baron R., Kneissel M. (2013). WNT signaling in bone homeostasis and disease: from human mutations to treatments. Nat. Med..

[6] Cosman F., Crittenden D.B., Adachi J.D., Binkley N., Czerwinski E., Ferrari S., Hofbauer L.C., Lau E., Lewiecki E.M., Miyauchi A. (2016). Romosozumab treatment in postmenopausal women with osteoporosis. N. Engl. J. Med..

[7] Li J., Sarosi I., Cattley R.C., Pretorius J., Asuncion F., Grisanti M., Morony S., Adamu S., Geng Z., Qiu W. (2006). Dkk1-mediated inhibition of Wnt signaling in bone results in osteopenia. Bone.

[8] Colditz J., Thiele S., Baschant U., Niehrs C., Bonewald L.F., Hofbauer L.C., Rauner M. (2018). Postnatal skeletal deletion of Dickkopf-1 increases bone formation and bone volume in male and female mice, despite increased sclerostin expression. J. Bone Miner. Res..

[9] Mukhopadhyay M., Shtrom S., Rodriguez-Esteban C., Chen L., Tsukui T., Gomer L., Dorward D.W., Glinka A., Grinberg A., Huang S.P. (2001). Dickkopf1 is required for embryonic head induction and limb morphogenesis in the mouse. Dev. Cell.

[10] Morvan F., Boulukos K., Clement-Lacroix P., Roman Roman S., Suc-Royer I., Vayssiere B., Ammann P., Martin P., Pinho S., Pognonec P. (2006). Deletion of a single allele of the Dkk1 gene leads to an increase in bone formation and bone mass. J. Bone Miner. Res..

[11] Tian E., Zhan F., Walker R., Rasmussen E., Ma Y., Barlogie B., Shaughnessy J.D. (2003). The role of the Wnt-signaling antagonist DKK1 in the development of osteolytic lesions in multiple myeloma. N. Engl. J. Med..

[12] Rauner M., Thiele S., Sinningen K., Winzer M., Salbach-Hirsch J., Gloe I., Peschke K., Haegeman G., Tuckermann J.P., Hofbauer L.C. (2013). Effects of the selective glucocorticoid receptor modulator compound A on bone metabolism and inflammation in male mice with collagen-induced arthritis. Endocrinology.

[13] Wang F.S., Ko J.Y., Yeh D.W., Ke H.C., Wu H.L. (2008). Modulation of Dickkopf-1 attenuates glucocorticoid induction of osteoblast apoptosis, adipocytic differentiation, and bone mass loss. Endocrinology.

[14] Ahmed S.F., Fouda N., Abbas A.A. (2013). Serum dickkopf-1 level in postmenopausal females: correlation with bone mineral density and serum biochemical markers. J. Osteoporos..

[15] Robling A.G., Niziolek P.J., Baldridge L.A., Condon K.W., Allen M.R., Alam I., Mantila S.M., Gluhak-Heinrich J., Bellido T.M., Harris S.E. (2008). Mechanical stimulation of bone in vivo reduces osteocyte expression of Sost/sclerostin. J. Biol. Chem..

[16] Kotiya A.A., Bayly P.V., Silva M.J. (2011). Short-term low-strain vibration enhances chemo-transport yet does not stimulate osteogenic gene expression or cortical bone formation in adult mice. Bone.

[17] Chan T.F., Couchourel D., Abed E., Delalandre A., Duval N., Lajeunesse D. (2011). Elevated Dickkopf-2 levels contribute to the abnormal phenotype of human osteoarthritic osteoblasts. J. Bone Miner. Res..

[18] Li X., Liu P., Liu W., Maye P., Zhang J., Zhang Y., Hurley M., Guo C., Boskey A., Sun L. (2005). Dkk2 has a role in terminal osteoblast differentiation and mineralized matrix formation. Nat. Genet..

[19] Van der Horst G., van der Werf S.M., Farih-Sips H., van Bezooijen R.L., Lowik C.W., Karperien M. (2005). Downregulation of Wnt signaling by increased expression of Dickkopf-1 and -2 is a prerequisite for late-stage osteoblast differentiation of KS483 cells. J. Bone Miner. Res..

[20] Li L., Mao J., Sun L., Liu W., Wu D. (2002). Second cysteine-rich domain of Dickkopf-2 activates canonical Wnt signaling pathway via LRP-6 independently of dishevelled. J. Biol. Chem..

[21] Chen L., Wang K., Shao Y., Huang J., Li X., Shan J., Wu D., Zheng J.J. (2008). Structural insight into the mechanisms of Wnt signaling antagonism by Dkk. J. Biol. Chem..

[22] Rudnicki M.A., Williams B.O. (2015). Wnt signaling in bone and muscle. Bone.

[23] Tajbakhsh S., Borello U., Vivarelli E., Kelly R., Papkoff J., Duprez D., Buckingham M., Cossu G. (1998). Differential activation of Myf5 and MyoD by different Wnts in explants of mouse paraxial mesoderm and the later activation of myogenesis in the absence of Myf5. Development.

[24] Munsterberg A.E., Kitajewski J., Bumcrot D.A., McMahon A.P., Lassar A.B. (1995). Combinatorial signaling by Sonic hedgehog and Wnt family members induces myogenic bHLH gene expression in the somite. Genes Dev..

[25] Otto A., Schmidt C., Luke G., Allen S., Valasek P., Muntoni F., Lawrence-Watt D., Patel K. (2008). Canonical Wnt signalling induces satellite-cell proliferation during adult skeletal muscle regeneration. J. Cell Sci..

[26] Murphy M.M., Keefe A.C., Lawson J.A., Flygare S.D., Yandell M., Kardon G. (2014). Transiently active Wnt/β-catenin signaling is not required but must be silenced for stem cell function during muscle regeneration. Stem Cell Rep..

[27] Kawao N., Morita H., Obata K., Tamura Y., Okumoto K., Kaji H. (2016). The vestibular system is critical for the changes in muscle and bone induced by hypergravity in mice. Physiol. Rep..

[28] Juffer P., Jaspers R.T., Lips P., Bakker A.D., Klein-Nulend J. (2012). Expression of muscle anabolic and metabolic factors in mechanically loaded MLO-Y4 osteocytes. Am. J. Physiol. Endocrinol. Metab..

[29] Guan P.P., Guo J.W., Yu X., Wang Y., Wang T., Konstantopoulos K., Wang Z.Y., Wang P. (2015). The role of cyclooxygenase-2, interleukin-1β and fibroblast growth factor-2 in the activation of matrix metalloproteinase-1 in sheared-chondrocytes and articular cartilage. Sci. Rep..

[30] Mao B., Niehrs C. (2003). Kremen2 modulates Dickkopf2 activity during Wnt/LRP6 signaling. Gene.

[31] Kuroda K., Kuang S., Taketo M.M., Rudnicki M.A. (2013). Canonical Wnt signaling induces BMP-4 to specify slow myofibrogenesis of fetal myoblasts. Skelet. Muscle.

[32] Hutcheson D.A., Zhao J., Merrell A., Haldar M., Kardon G. (2009). Embryonic and fetal limb myogenic cells are derived from developmentally distinct progenitors and have different requirements for β-catenin. Genes Dev..

[33] Yin J., Yang L., Xie Y., Liu Y., Li S., Yang W., Xu B., Ji H., Ding L., Wang K. (2018). Dkk3 dependent transcriptional regulation controls age related skeletal muscle atrophy. Nat. Commun..

[34] Han S.O., Kommaddi R.P., Shenoy S.K. (2013). Distinct roles for β-arrestin2 and arrestin-domain-containing proteins in β2 adrenergic receptor trafficking. EMBO Rep..

[35] Gordon B.S., Rossetti M.L., Eroshkin A.M. (2019). Arrdc2 and Arrdc3 elicit divergent changes in gene expression in skeletal muscle following anabolic and catabolic stimuli. Physiol. Genomics.

[36] Furlow J.D., Watson M.L., Waddell D.S., Neff E.S., Baehr L.M., Ross A.P., Bodine S.C. (2013). Altered gene expression patterns in muscle ring finger 1 null mice during denervation- and dexamethasone-induced muscle atrophy. Physiol. Genomics.

[37] Kumar A., Tikoo S., Maity S., Sengupta S., Kaur A., Bachhawat A.K. (2012). Mammalian proapoptotic factor ChaC1 and its homologues function as γ-glutamyl cyclotransferases acting specifically on glutathione. EMBO Rep..

[38] Deloux R., Tannous C., Ferry A., Li Z., Mericskay M. (2018). Aged nicotinamide riboside kinase 2 deficient mice present an altered response to endurance exercise training. Front. Physiol..

[39] Krupnik V.E., Sharp J.D., Jiang C., Robison K., Chickering T.W., Amaravadi L., Brown D.E., Guyot D., Mays G., Leiby K. (1999). Functional and structural diversity of the human Dickkopf gene family. Gene.

[40] Niehrs C. (2006). Function and biological roles of the Dickkopf family of Wnt modulators. Oncogene.

[41] Vervloet M.G., Massy Z.A., Brandenburg V.M., Mazzaferro S., Cozzolino M., Urena-Torres P., Bover J., Goldsmith D. (2014). Bone: a new endocrine organ at the heart of chronic kidney disease and mineral and bone disorders. Lancet. Diabetes Endocrinol..

[42] Banzrai C., Nodera H., Kawarai T., Higashi S., Okada R., Mori A., Shimatani Y., Osaki Y., Kaji R. (2016). Impaired axonal Na^+^ current by hindlimb unloading: implication for disuse neuromuscular atrophy. Front. Physiol..

[43] Yano M., Kawao N., Okumoto K., Tamura Y., Okada K., Kaji H. (2014). Fibrodysplasia ossificans progressiva-related activated activin-like kinase signaling enhances osteoclast formation during heterotopic ossification in muscle tissues. J. Biol. Chem..

[44] Ishida M., Kawao N., Okada K., Tatsumi K., Sakai K., Nishio K., Kaji H. (2018). Serpina3n, dominantly expressed in female osteoblasts, suppresses the phenotypes of differentiated osteoblasts in mice. Endocrinology.

[45] Bouxsein M.L., Boyd S.K., Christiansen B.A., Guldberg R.E., Jepsen K.J., Muller R. (2010). Guidelines for assessment of bone microstructure in rodents using micro-computed tomography. J. Bone Miner. Res..

[46] Kawao N., Ishida M., Kaji H. (2019). Roles of leptin in the recovery of muscle and bone by reloading after mechanical unloading in high fat diet-fed obese mice. PLoS ONE.

[47] Kawao N., Tamura Y., Okumoto K., Yano M., Okada K., Matsuo O., Kaji H. (2013). Plasminogen plays a crucial role in bone repair. J. Bone Miner. Res..

[48] Kawao N., Tamura Y., Okumoto K., Yano M., Okada K., Matsuo O., Kaji H. (2014). Tissue-type plasminogen activator deficiency delays bone repair: roles of osteoblastic proliferation and vascular endothelial growth factor. Am. J. Physiol. Endocrinol. Metab..

