# Supplementary material for: Roles of Dkk2 in the Linkage from Muscle to Bone during Mechanical Unloading in Mice
Source: Int J Mol Sci. 2020 Apr 6;21(7):2547. doi: 10.3390/ijms21072547 (PMC7177709; doi:10.3390/ijms21072547)
Supplement: Supplementary file 1 [file ijms-21-02547-s001.zip › Dkk2_TableS2primer.docx]

**Table S2** Primers used for real-time PCR experiments.

| Gene |  | Primer sequence |
| --- | --- | --- |
| Dkk1 | Forward  Reverse | 5’-GCCTCCGATCATCAGACGGT-3’  5’-GCAGGTGTGGAGCCTAGAAG-3’ |
| Dkk2 | Forward  Reverse | 5’-CTGATGCGGGTCAAGGATTCA-3’  5’-CTCCCCTCCTAGAGAGGACTT-3’ |
| Dkk3 | Forward  Reverse | 5’-GAGATGTTTCGAGAGGTGGAG-3’  5’-TTGTGATAGTTGGGAGGTAAGC-3’ |
| Dkk4  Sclerostin | Forward  Reverse  Forward  Reverse | 5’-CTGTGTGAATGATGTTTGCAC-3’  5’-GTCAGAGGTTCTAAGACAGC-3’  5’-CTACTTGTGCACGCTGCCTT-3’  5’-TTTGGCGTCATAGGGATGGT-3’ |
| Sfrp1 | Forward  Reverse | 5’-CAGGTCTTCCTCTGTT-3’  5’-GGCTTCCGTGGTATTG-3’ |
| Sfrp2 | Forward  Reverse | 5’-ATCGAGTACCAGAACA-3’  5’-CGTCGAGACAGACAGG-3’ |
| Sfrp3 | Forward  Reverse | 5’-CAAGGGACACCGTCAATCTT-3’  5’-CATATCCCAGCGCTTGACTT-3’ |
| Sfrp4 | Forward  Reverse | 5’-ACCTGAGCAAAAACTACAGCTATG-3’  5’-CTACCACAGTTGTGACCTCATTG-3’ |
| Sfrp5 | Forward  Reverse | 5’-GAAAGTTGATTGGAGCCCAGAA-3’  5’-GCCCGTCAGGTTGTCTAACTGT-3’ |
| MyoD | Forward | 5’-AGCACTACAGTGGCGACTCAG-3’ |
|  | Reverse | 5’-AGGCGGTGTCGTAGCCATTC-3’ |
| Myogenin | Forward | 5’-GCTGCCTAAAGTGGAGATCCT-3’ |
|  | Reverse | 5’-GCGCTGTGGGAGTTGCAT-3’ |
| MHC-I | Forward | 5’-GCCAACTATGCTGGAGCTGATGCCC-3’ |
|  | Reverse | 5’-GGTGCGTGGAGCGCAAGTTTGTCATAAG-3’ |
| Pax7 | Forward | 5’-CCCTCAGTGAGTTCGATTAGCC-3’ |
|  | Reverse | 5’-GGTCGGGTTCTGATTCCACA-3’ |
| Atrogin-1 | Forward | 5’-GTCGCAGCCAAGAAGAGAAAGA-3’ |
|  | Reverse | 5’-TGCTATCAGCTCCAACAGCCTT-3’ |
| Runx2 | Forward  Reverse | 5’-AAATGCCTCCGCTGTTATGAA-3’  5’-GCTCCGGCCCACAAATCT-3’ |
| Osterix | Forward  Reverse | 5’-AGCGACCACTTGAGCAAACAT-3’  5’-GCGGCTGATTGGCTTCTTCT-3’ |
| ALP | Forward  Reverse | 5’-ATCTTTGGTCTGGCTCCCATG-3’  5’-TTTCCCGTTCACCGTCCAC-3’ |
| Osteocalcin | Forward  Reverse | 5’-CCTGAGTCTGACAAAGCCTTCA-3’  5’-GCCGGAGTCTGTTCACTACCTT-3’ |
| RANKL | Forward | 5’-CACAGCGCTTCTCAGGAGCT-3’ |
|  | Reverse | 5’-CATCCAACCATGAGCCTTCC-3’ |
| OPG | Forward | 5’-**AGTCCGTGAAGCAGGAGT**-3’ |
|  | Reverse | 5’-**CCATCTGGACATTTTTTGCAAA**-3’ |
| GAPDH | Forward | 5’-AGGTCGGTGTGAACGGATTTG-3’ |
|  | Reverse | 5’-GGGGTCGTTGATGGCAACA-3’ |

Dkk, Dickkopf; Sfrp, secreted frizzled related protein; MHC, myosin heavy chain; ALP, alkaline phosphatase; RANKL, receptor activator of nuclear factor κB ligand; OPG, osteoprotegerin; GAPDH, glyceraldehyde-3-phosphate dehydrogenase.
