# Supplementary material for: Roles of Dkk2 in the Linkage from Muscle to Bone during Mechanical Unloading in Mice
Source: Int J Mol Sci. 2020 Apr 6;21(7):2547. doi: 10.3390/ijms21072547 (PMC7177709; doi:10.3390/ijms21072547)
Supplement: Supplementary file 1 [file ijms-21-02547-s001.zip › DKK2_TableS1.docx]

**Table S1** Correlation between Dkk2 levels and levels of osteoblastic differentiation-related genes in the tibia of control mice and mice with HU.

|  | Serum Dkk2 | |  | Soleus muscle  Dkk2 mRNA levels | |
| --- | --- | --- | --- | --- | --- |
|  | r | *P* |  | r | *P* |
| Runx2 | -0.159 | 0.558 |  | 0.363 | 0.167 |
| Osterix | -0.201 | 0.455 |  | 0.425 | 0.101 |
| ALP | -0.335 | 0.204 |  | 0.171 | 0.526 |
| Osteocalcin | 0.054 | 0.843 |  | 0.051 | 0.851 |

Simple regression analysis was performed in serum Dkk2 or soleus Dkk2 mRNA levels and mRNA levels of Runx2, Osterix, alkaline phosphatase (ALP) and osteocalcin in the tibia of control mice and mice with HU.
